# The CellBox-2 Mission to the International Space Station: Thyroid Cancer Cells in Space

**DOI:** 10.3390/ijms22168777

**Published:** 2021-08-16

**Authors:** Daniela Melnik, Marcus Krüger, Herbert Schulz, Sascha Kopp, Markus Wehland, Johann Bauer, Bjorn Baselet, Randy Vermeesen, Sarah Baatout, Thomas J. Corydon, Manfred Infanger, Daniela Grimm

**Affiliations:** 1 Department of Microgravity and Translational Regenerative Medicine, Otto von Guericke University, Universitätsplatz 2, 39106 Magdeburg, Germany; daniela.melnik@med.ovgu.de (D.M.); marcus.krueger@med.ovgu.de (M.K.); herbert.schulz@med.ovgu.de (H.S.); sascha.kopp@med.ovgu.de (S.K.); markus.wehland@med.ovgu.de (M.W.); manfred.infanger@med.ovgu.de (M.I.); 2 Research Group ‘Magdeburger Arbeitsgemeinschaft für Forschung unter Raumfahrt-und Schwerelosigkeitsbedingungen’ (MARS), Otto von Guericke University, Universitätsplatz 2, 39106 Magdeburg, Germany; 3 SiHaTho GmbH, Pferdsbach 4a, 35216 Biedenkopf, Germany; jbauer@sihatho.de; 4 SCK CEN, Belgian Nuclear Research Centre, Boeretang 200, 2400 Mol, Belgium; bjorn.baselet@sckcen.be (B.B.); randy.vermeesen@sckcen.be (R.V.); sarah.baatout@sckcen.be (S.B.); 5 Department Molecular Biotechnology, Gent University, 9000 Gent, Belgium; 6 Department of Biomedicine, Aarhus University, Ole Worms Allé 4, 8000 Aarhus, Denmark; corydon@biomed.au.dk; 7 Department of Ophthalmology, Aarhus University Hospital, Palle Juul-Jensens Boulevard 167, 8200 Aarhus, Denmark

**Keywords:** spaceflight, thyroid cancer, growth, spheroids, focal adhesion, cytokines, growth factors, cell signalling, International Space Station, extracellular matrix

## Abstract

A spaceflight to the International Space Station (ISS) is a dream of many researchers. We had the chance to investigate the effect of real microgravity (CellBox-2 Space mission) on the transcriptome and proteome of FTC-133 human follicular thyroid cancer cells (TCC). The cells had been sent to the ISS by a Falcon 9 rocket of SpaceX CRS-13 from Cape Canaveral (United States) and cultured in six automated hardware units on the ISS before they were fixed and returned to Earth. Multicellular spheroids (MCS) were detectable in all spaceflight hardware units. The *VCL*, *PXN*, *ITGB1*, *RELA*, *ERK1* and *ERK*2 mRNA levels were significantly downregulated after 5 days in space in adherently growing cells (AD) and MCS compared with ground controls (1*g*), whereas the *MIK67* and *SRC* mRNA levels were both suppressed in MCS. By contrast, the *ICAM1*, *COL1A1* and *IL6* mRNA levels were significantly upregulated in AD cells compared with 1*g* and MCS. The protein secretion measured by multianalyte profiling technology and enzyme-linked immunosorbent assay (AngiogenesisMAP^®^, extracellular matrix proteins) was not significantly altered, with the exception of elevated angiopoietin 2. TCC in space formed MCS, and the response to microgravity was mainly anti-proliferative. We identified ERK/RELA as a major microgravity regulatory pathway.

## 1. Introduction

The Global Cancer Observatory (Globocan) 2020 estimated that thyroid cancer (TC) is responsible for 586,000 cases of cancer worldwide [1]. Women have a 3-fold higher global incidence rate than men [1]. The American Cancer Society recently estimated that in the United States, there will be about 44,280 new cases of TC (12,150 in men and 32,130 in women) and about 2200 deaths from TC (1050 men and 1150 women) in 2021 [2]. The number of deaths was slightly elevated from 2009 to 2018 (0.6% per year) but seems to have been stable in recent years.

TC is classified into several categories: (1) differentiated TC, covering papillary, follicular and Hürthle cell cancer, (2) medullary and (3) anaplastic [3]. In general, patients with differentiated TC have a long-term survival rate near 90%. By contrast, the poorly differentiated TC types show a more discouraging long-term survival rate of close to 10%, which is a result of their resistance to the standard treatment options [4,5,6]. Treatment options for advanced and RAI-refractory TC are multi-kinase inhibitors, immunotherapy and chemotherapeutic drugs [4,7]. In general, the survival rate is still poor and therefore novel research approaches are necessary.

A new idea to increase our knowledge in cancer research is to perform studies in microgravity. Microgravity conditions occurring during space missions impact the morphology, function, gene expression and protein synthesis of normal thyroid cells and thyroid cancer cells (TCC) [8]. TC is one of the most common carcinoma types of the endocrine organs [9]. To study additional characteristics of TC, primary cultures and cell lines have been derived from thyroid tumours [10,11,12]. The TCC lines have been used as a model to study the influence of real microgravity (r-µ*g*) and simulated microgravity (s-µ*g*). A large number of cells of different cancer types have been exposed to microgravity conditions to learn more about changes in biological processes such as cell adhesion, spreading of cancer cells and metastasis [13,14].

Poorly differentiated follicular TCC (FTC-133, ML-1, UCLA RO82-W-1) have been exposed to s-µ*g* conditions created by a random positioning machine (RPM) or a clinostat [14,15,16,17]. These in vitro studies have shown that s-µ*g* induces a variety of alterations in human TCC, such as changes in proliferation, cell signalling, differentiation and gene expression, and it triggers scaffold-free formation of multicellular spheroids (MCS). Moreover, studies on cells exposed to r-µ*g* for a short time, as performed by means of TEXUS (TX) sounding rocket missions (6 min) and parabolic flights (22 s), have demonstrated changes in the structure and organisation of the cytoskeleton as well as alterations in gene expression patterns [18,19]. Using a suitable hardware, TCC have been investigated in space during three different spaceflights, each lasting about 10 days [20,21,22]. After the SimBox/Shenzhou-8 spaceflight, there were spheroids of considerable size that could be observed as well as important and significant shifts in the gene expression pattern [22,23]. However, no spheroid formation was observed during the CellBox-1 mission. Interestingly, in this mission, there was detected an accumulation of caveolin-1 [21,24]. This discrepancy could be explained by a longer delay in the rocket launch and the simultaneous formation of a confluent cell monolayer. 

To determine the impact of r-µ*g* (spaceflight) on TCC, the CellBox-2 mission was performed [20]. In this mission to the International Space Station (ISS) flown in December 2017, a lower temperature (28 °C) during the pre-launch phase avoided the formation of a confluent cell monolayer, despite some delay in the launch. After returning from the ISS mission, the low-differentiated follicular TCC cultures had formed small spheroids. These cultures were used to further characterise gene expression changes of low-differentiated follicular TCC and their secretory behaviour of various proteins of interest during a spaceflight compared with normal culturing of the same cells on Earth in the flight hardware.

## 2. Results

The FTC-133 cells returning from the CellBox-2 space mission to the ISS were harvested and characterised as described previously [20]. To gain more information about the behaviour of these cells in space, we used the cultures from the CellBox-2 mission and focused on additional changes in gene expression patterns and measured proteins released into the supernatants during a 5- or 10-day stay in orbit (Table 1). Adherent cells (AD) and MCS harvested from the automated hardware units (space-suitable culture chambers (SSCC)) were analysed by quantitative real-time polymerase chain reaction (qPCR), applying the primers listed in Table 2.

### 2.1. Impact of Microgravity on Selected Genes

**Figure 1:**
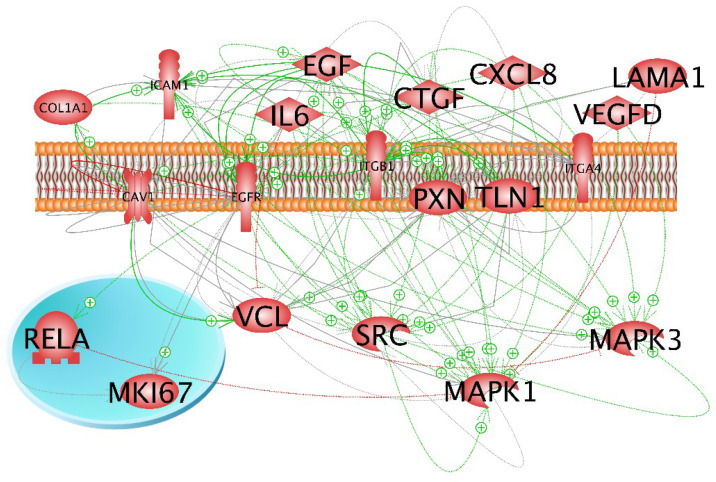
Localisation and interaction of the gene products selected for quantitative real-time polymerase chain reaction analysis. The blue circle indicates the nucleus, and the orange structure shows the membrane. Green arrows indicate stimulation and red lines with a terminal crossbar show inhibition. Grey arrows indicate interactions with an unknown effect. Grey lines show protein–protein complex formation. Solid arrows indicate regulation by direct interaction and dashed arrows indicate indirect regulation via other cell components.

**Figure 2:**
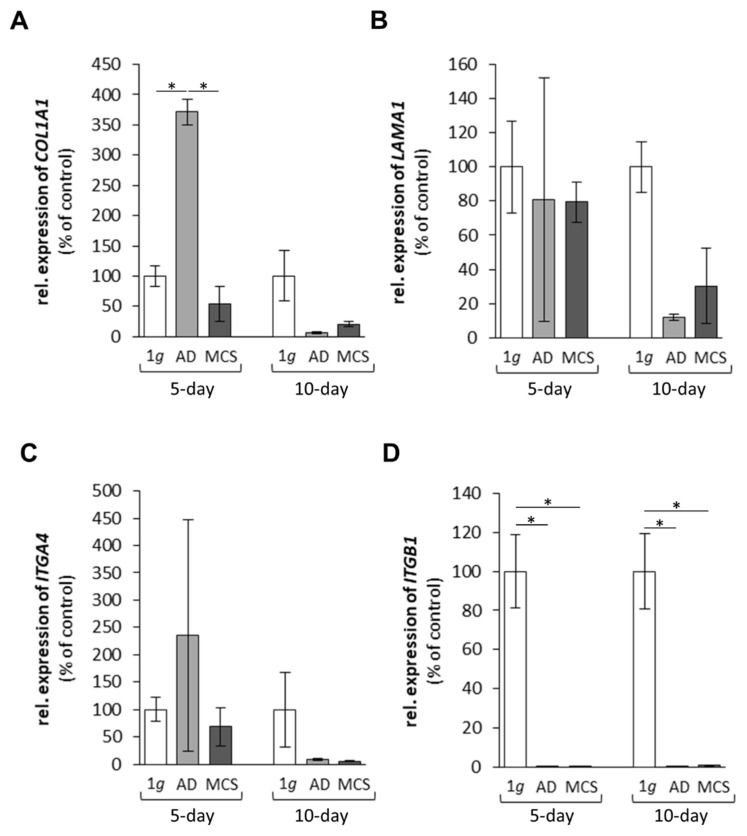
Gene expression of (**A**) *COL1A1*, (**B**) *LAMA1*, (**C**) *ITGA4*, and (**D**) *ITGB1* of FTC-133 follicular thyroid cancer cells grown in space on the International Space Station for 5 and 10 days. * *p* < 0.05 versus 1*g* ground control cells. Values are presented as the mean ± standard deviation in percent of 1*g* ground controls, which was fixed as 100% for each condition. AD corresponds to adherent cells; MCS corresponds to multicellular spheroids. 5-day spaceflight/1*g* ground control samples, *n* = 3; 10-day spaceflight samples/1*g* ground control, *n* = 2.

**Figure 3:**
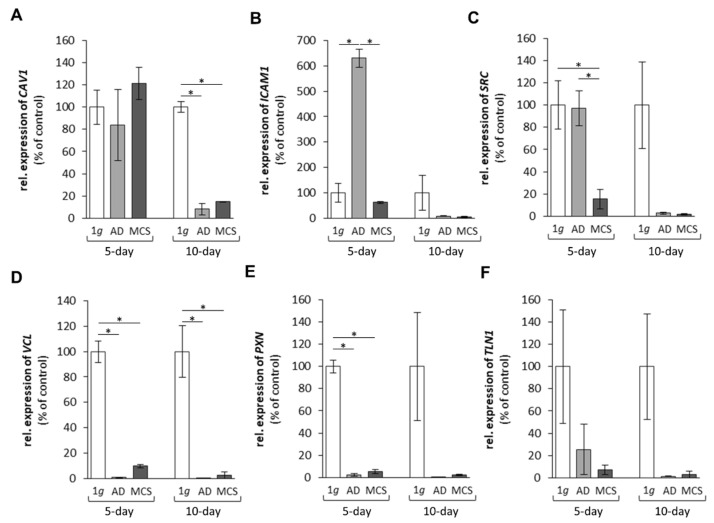
Gene expression of (**A**) *CAV1*, (**B**) *ICAM1*, (**C**) *SRC*, (**D**) *VCL*, (**E**) *PXN* and (**F**) *TLN1* of FTC-133 follicular thyroid cancer cells grown in space on the International Space Station for 5 and 10 days. * *p* < 0.05 versus 1*g* ground control cells. Values are presented as the mean ± standard deviation in percent of 1*g* ground controls, which was fixed as 100% for each condition. AD corresponds to adherent cells; MCS corresponds to multicellular spheroids. 5-day spaceflight/1*g* ground control samples, *n* = 3; 10-day spaceflight samples/1*g* ground control, *n* = 2.

**Figure 4:**
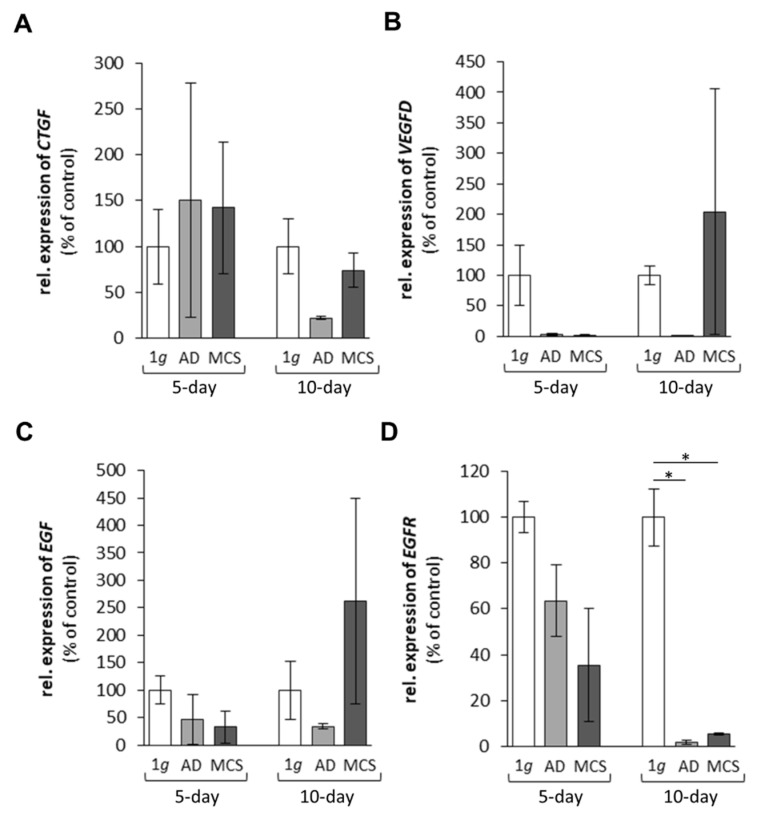
Gene expression of (**A**) *CTGF*, (**B**) *VEGFD*, (**C**) *EGF* and (**D**) *EGFR* of FTC-133 follicular thyroid cancer cells grown in space on the International Space Station for 5 and 10 days. * *p* < 0.05 versus 1*g* ground control cells. Values are presented as the mean ± standard deviation in percent of 1*g* ground controls, which was fixed as 100% for each condition. AD corresponds to adherent cells; MCS corresponds to multicellular spheroids. 5-day spaceflight/1*g* ground control samples, *n* = 3; 10-day spaceflight samples/1*g* ground control, *n* = 2.

**Figure 5:**
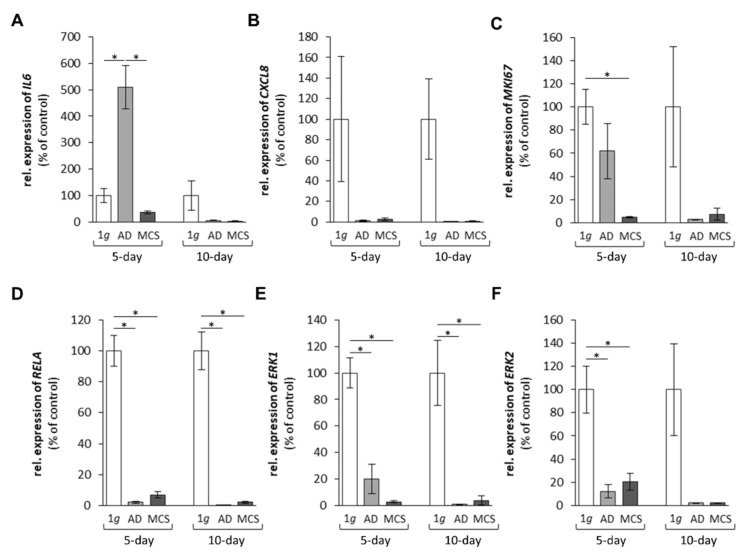
Gene expression of (**A**) *IL6*, (**B**) *CXCL8*, (**C**) *MKI67*, (**D**) *RELA*, (**E**) *ERK1* and (**F**) *ERK2* of FTC-133 follicular thyroid cancer cells grown in space on the International Space Station for 5 and 10 days. * *p* < 0.05 versus 1*g* ground control cells. Values are presented as the mean ± standard deviation in percent of 1*g* ground controls, which was fixed as 100% for each condition. AD corresponds to adherent cells; MCS corresponds to multicellular spheroids. 5-day spaceflight/1*g* ground control samples, *n* = 3; 10-day spaceflight samples/1*g* ground control, *n* = 2.

In a first approach, we investigated the expression of selected genes, which had demonstrated gravi-sensitivity in earlier studies [18,23]. These genes code for proteins, which are located within the cytoplasm, the membrane and the extracellular space (Figure 1), and form a network of interaction, connecting extracellular factors via membrane proteins with intracellular signalling factors and nuclear proteins. The qPCR results are provided in Figure 2, Figure 3, Figure 4 and Figure 5. Applying qPCR, the genes of the proteins shown in Figure 1 were investigated with regard to the quantities of their mRNA transcripts expressed at the time of harvest. 

#### 2.1.1. Genes Encoding Extracellular Matrix Proteins

The gene expression of *COL1A1* was significantly enhanced in AD cells versus 1*g* ground control cells and MCS after 5 days in space (Figure 2A). After 10 days in space, the *COL1A1* gene expression in AD cells and MCS was low (Figure 2A). In addition, we measured the *LAMA1* mRNA after 5 and 10 days. There was no significant change in *LAMA1* in all groups (Figure 2B). A similar result was obtained for the *ITGA4* gene, which was not changed in all groups (Figure 2C). By contrast, the *ITGB1* mRNA was significantly reduced in all spaceflight samples after 5 and 10 days compared with the corresponding 1*g* ground controls (Figure 2D).

#### 2.1.2. Genes Encoding for Membrane Proteins Involved in Cell Adhesion

We focused on selected genes involved in cell adhesion. First, we measured caveolin-1 encoded by *CAV1*. After 5 days in space, the cells exhibited no change in the *CAV1* gene expression. After 10 days in space, there was a significant downregulation of *CAV1* in AD cells and MCS compared with 1*g* samples (Figure 3A). Second, we investigated the intercellular adhesion molecule 1 in humans encoded by the *ICAM1* gene. After 5 days, there was a clear upregulation of *ICAM1* in AD cells compared with 1*g* samples and MCS. After 10 days, the *ICAM1* mRNA was not different among the groups (Figure 3B).

The proto-oncogene tyrosine-protein kinase Src in humans is encoded by the *SRC* gene. The *SRC* mRNA was significantly downregulated in MCS after 5 days and blunted in AD cells and MCS after 10 days compared with 1*g* cells (Figure 3C). The cell adhesion molecule vinculin encoded by the *VCL* gene was significantly downregulated in AD cells and MCS after 5 and 10 days compared with the corresponding 1*g* ground controls (Figure 3D). A similar result was obtained for paxillin, which is encoded by *PXN* in humans. The mRNA was downregulated in AD cells and MCS after 5 days in space (Figure 3E). No significant change was found for talin-1 (*TLN1*) in the samples (Figure 3F).

#### 2.1.3. Genes Encoding for Factors Promoting Angiogenesis and Tumour Growth 

The connective tissue growth factor (*CTGF*) mRNA expression during spaceflight was not significantly changed in 5- and 10-day r-µ*g* samples (Figure 4A). The vascular endothelial growth factor D (*VEGFD*) gene expression was not significantly changed after 5 days and 10 days in AD cells and MCS compared with 1*g* (Figure 4B). A similar result was obtained for the epidermal growth factor (*EGF*) gene expression which was not differentially regulated in the spaceflight samples (Figure 4C). 

By contrast, the *EGFR* mRNA was unaltered after 5 days, but significantly downregulated after 10 days of spaceflight in AD cells and MCS (Figure 4D). 

#### 2.1.4. Cytokines and the Extracellular Signal-Regulated Kinases

The interleukin-6 (*IL6*) mRNA expression was enhanced in AD cells compared with the corresponding 1*g* ground control samples after 5 days, but not significantly regulated in spaceflight samples after 10 days (Figure 5A). The interleukin-8 (*IL8*; also called C-X-C motif chemokine ligand 8 (*CXCL8*)) mRNA expression was lower in all spaceflight samples at both time points compared with 1*g* ground control samples (Figure 5B). In addition, the marker of proliferation *KI67* (also known as *MKI67*) was significantly reduced in MCS after 5 days on the ISS, but not significantly changed after 10 days (Figure 5C). The nuclear factor of kappa light polypeptide gene enhancer in B-cells (*RELA*) gene expression was downregulated in AD and MCS spaceflight samples after 5 and 10 days (Figure 5D). Moreover, the extracellular signal-regulated kinases 1 (*ERK1*) and 2 (*ERK2*) showed a very similar, clear downregulation after 5 and 10 days of spaceflight (Figure 5E,F) compared with 1*g*.

### 2.2. Impact of Microgravity on the Secretion of Proteins

#### Secreted Factors Involved in Angiogenesis

Tumour growth and development depends on neoangiogenesis. Therefore, we applied the AngiogenesisMAP^®^ of Myriad RBM technology for our search. We focused on the quantities of proteins involved in neovascularisation detectable in the supernatants of FTC-133 cells cultured for 5 or 10 days in SSCC under r-µ*g* on the ISS and in our laboratory on the ground in SSCC as corresponding 1*g* ground control*s*.

Of the factors that can be measured in principle by AngiogenesisMAP^®^ analysis, the following 32 proteins were not detectable: Angiopoetin-1, CKine, collagen IV, decorin, endoglin, hepsin, Her-2, insulin-like growth factor-binding protein 2, carcinoembryonic antigen-related cell adhesion molecule 1, insulin-like growth factor-binding protein 2, interferon gamma, interferon-inducible T-cell alpha chemoattractant, interleukin-1 alpha, interleukin-1 beta, interleukin-2, interleukin-10, kallikrein-5, kallikrein-7, macrophage inflammatory protein 1-alpha, macrophage inflammatory protein 3-beta, maspin, matrix metalloproteinase-9, platelet endothelial cell adhesion molecule, platelet-derived growth factor BB, tumour necrosis factor alpha, tumour necrosis factor beta, vascular endothelial growth factor receptor 1, vascular endothelial growth factor receptor 2, vascular endothelial growth factor receptor 3, interleukin-1 alpha, stem cell factor and stromal cell-derived factor-1.

The 19 factors secreted by the FTC-133 cells in the AngiogenesisMAP^®^ experiment and the six factors secreted by the FTC-133 cells in ELISA that were measurable in space and/or on Earth are listed in Table 1. 

Of the 25 factors, seventeen analytes had a sufficient concentration in all samples and has been submitted to one-way ANOVA. The only released factor which was significantly altered by spaceflight was angiopoetin-2.

Cancer antigen 15-3 (CA-15-3) was only detectable in 10-day flight supernatants, and it was below the lower limit of quantitation (LLOQ) in all other groups. Interestingly, fatty acid-binding protein, adipocyte (FABP, adipocyte) was not released by FTC-133 cells grown under 1*g* conditions on Earth, but FABP was detectable in space after 5 and 10 days (Table 1). Granulocyte-macrophage colony-stimulating factor (GM-CSF) was not released by the FTC-133 ground control cells after 5 days, but the protein was detectable in space after 5 days. After a 10-day culture in space and on Earth, the cells secreted a similar amount of GM-CSF in the supernatant. FTC-133 cells secreted macrophage inflammatory protein-1 beta (MIP-1 beta) after 5 and 10 days at 1*g* conditions. After 5 days in space, the detectable amount of MIP-1 beta was similar to 1*g* ground controls. By contrast, FTC-133 did not release a detectable amount of MIP-1 beta in the supernatant after 10 days (Table 1). In addition, EGF was released in a detectable amount after 10 days under 1*g* ground control conditions, but the cells grown in space did not secrete a measurable amount. Insulin-like growth factor-binding protein 2 (IGFBP-2) was secreted by the FTC-133 cells in a similar and high amount in space and on Earth (Table 1). Placenta growth factor (PLGF) was not secreted by FTC-133 under normal static 1*g* conditions at 5 and 10 days, whereas PLGF was detectable after 5 days in space, but not after 10 days (Table 1). 

**Figure 6:**
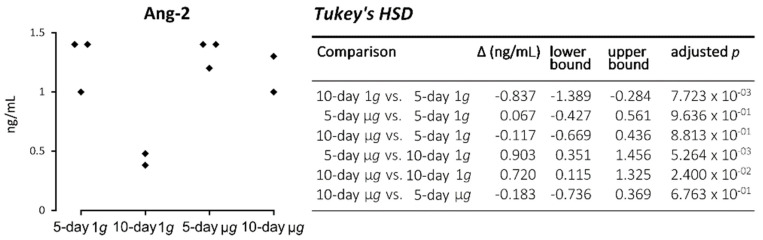
Angiopoetin-2 (Ang-2) MAP analytes concentration and statistical evaluation.

In addition, we focused on components of the extracellular matrix (ECM) released in the cell supernatants with the means of the Illumina enzyme-linked immunosorbent assay (ELISA) technology. We did not measure significant changes in the following ECM proteins: collagen I alpha I, laminin, fibronectin and osteopontin (Table 1). Furthermore, the secretion of the signalling elements lipocalin-2/NGAL and VEGF-D was not significantly altered (Table 1). The release of Ang-2 in the cell supernatant was not altered after 5 days in space. After 10 days, 1*g* ground control cells secreted a significantly decreased amount of Ang-2. By contrast, Ang-2 was significantly elevated in 10-day space samples (Figure 6).

## 3. Discussion

In this study, we investigated the behaviour of poorly differentiated follicular TCC in space on the ISS (the Cellbox-2 mission) and on Earth using the same hardware container and conditions as the spaceflight. We focused on factors known to be gravi-sensitive and involved in cancer angiogenesis, progression and metastasis. The cells cultured in space showed two different phenotypes: one part of the cells grew adherently, while the other part formed MCS during the spaceflight together with the stay on the ISS. This observation had been reported earlier during the Shenzhou-8/SimBox spaceflight experiment [22,23] and in studies using microgravity-simulating devices, such as clinostats or the RPM [15,16,25]. Gravitational unloading is known to influence several processes in cancer cells: differentiation, proliferation, apoptosis, growth, migration and invasion, among others [23,26]. The gene expression of a large number of genes in various cell types in vivo and in vitro is differentially altered in space compared to 1*g* conditions on Earth [23,27,28,29].

### 3.1. Changes in Gene Expression Patterns of Thyroid Cancer Cells in Microgravity

#### 3.1.1. Extracellular Matrix Proteins Altered by r-µ*g* Exposure

**Figure 7:**
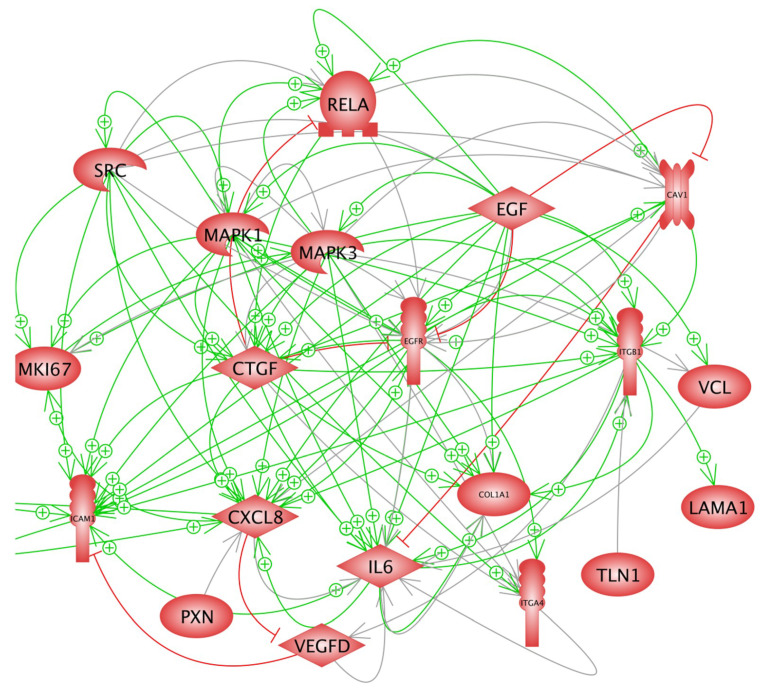
Interaction of the genes analysed by quantitative real-time polymerase chain reaction. Green arrows indicate stimulation and red lines with a terminal crossbar show inhibition. Grey arrows indicate an interaction with an unknown effect. Of note, most of the green arrows start near the SRC and EGF icons.

The gene expression of *COL1A1* was significantly upregulated in AD cells after 5 days in space compared with MCS and 1*g* ground control cells (Figure 2A), whereas after 10 days, there were no significant changes. EGF, MAPK1, MAPK3, CTGF and ITGB1 all activate COL1A1, as demonstrated in Figure 7, but *EGF* and *CTGF* were not significantly altered, and *ITGB1*, *ERK1* and *ERK2* were downregulated in space. COL1A1 is known to promote the migration and invasion of ovarian cancer cells in vitro [30]. The transient rise in the *COL1A1* mRNA level in AD cells in space hints to its involvement in cell detachment and aggregation to three-dimensional (3D) spheroids in the early phases of microgravity. FTC-133 TCC exposed for 7 and 14 days to an RPM showed a reduced *COL1A1* mRNA level in all RPM samples compared with the corresponding static 1*g* samples [31], and those findings are comparable to the results obtained after a 10-day r-µ*g* exposure in space. The secretion of collagen-1 and other ECM proteins in the cell supernatant was high but not altered in any of the groups (Table 1).

Furthermore, we focused on integrins, in particular β_1_-integrin. These ECM receptors are involved in cell adhesion and migration and are important for morphogenesis [32]. *ITGB1* was downregulated in all spaceflight samples at 5 and 10 days. A similar result was obtained when MCF-7 breast cancer cells were investigated during a parabolic flight mission [33]. β_1_-integrin protein was significantly reduced after the 1^st^ and 31^st^ parabola [33]. The downregulation of β_1_-integrin may inhibit cell adhesion to the ECM. In addition, MCF-7 breast cancer cells exposed to the rotating wall vessel (RWV) showed a decrease in β_1_-integrin protein [34], supporting the recent finding that focal adhesion is reduced in space and under s-µ*g* conditions [35].

#### 3.1.2. Cell Adhesion Factors Changed in Space

Warnke et al. [15] had demonstrated a downregulation in the *CAV1* gene when poorly differentiated thyroid FTC-133 TCC formed spheroids under s-µg conditions using a two-dimensional (2D) clinostat and an RPM. Here, we demonstrated a downregulation in the *CAV1* mRNA level after 10 days in space. Interestingly, after 5 days, the *CAV1* gene was not significantly changed (Figure 3). Earlier, we had shown that the caveolin-1 protein is involved in the inhibition of spheroid formation when confluent monolayers are exposed to microgravity [21,24]. This result confirms the new results obtained from spaceflight samples and the earlier data obtained from microgravity simulators. Caveolin-1 is a scaffold protein and known to interact in several pathways, including integrin signalling [36]. In summary, there is evidence that caveolin-1 regulates 3D growth in poorly differentiated TCC grown in space.

ICAM-1 is known to be a gravi-sensitive element. Primary human M1 macrophages (ISS experiment, 11 days) showed a decreased ICAM-1 expression compared with 1*g* controls [37]. Similar findings were obtained when murine BV-2 microglial cells were exposed to clinorotation or parabolic flight manoeuvres. They exhibited downregulated ICAM-1 expression, specifically rapid and reversible downregulation in the microgravity phase of the parabola [38]. By contrast, macrophage-like differentiated human U937 cells presented an elevated expression of ICAM-1, when the cells were exposed to clinorotation, short-term (PF) or long-term r-µ*g* (SIMBOX/Shenzhou-8 mission) [38]. EA.hy926 endothelial cells grown for 3 and 10 days on board the SJ-10 Satellite showed decreased expression of the ICAM-1 protein [39]. Adherently growing FTC-133 TCC revealed an upregulation of the *ICAM1* mRNA after 5 days in space compared with 1*g* and MCS samples. After 10 days, the *ICAM1* gene was not significantly changed. This is in agreement with the results obtained with MDA-mb-231 triple-negative breast cancer cells exposed to parabolic flight manoeuvres, which exhibited an early upregulation of *ICAM1* [40]. The adhesion molecule ICAM-1 is associated with different cancer types. It is known to be involved in cancer metastasis and may serve as a biomarker and as a possible target for therapeutic interventions [41].

The proto-oncogene tyrosine-protein kinase Src is encoded by the *SRC* gene, which was significantly downregulated in MCS compared with AD and 1*g* samples. The product of the human *SRC* gene, c-Src, is found to be overexpressed and highly activated in a wide variety of human cancers and plays a key role in progression and metastasis. Src is elevated by direct or indirect interaction with EGFR [42]. Moreover, tyrosine kinome profiling demonstrated the elevation of nine tyrosine kinases in tumours compared with matched normal thyroid tissue: ‘*EGFR, PTK6, BTK, HCK, ABL1, TNK1, GRB2, ERK and SRC*’ [43]. Invasive disease revealed elevated Src activity in invasive tumours relative to non-invasive tumours [43].

The expression of the focal adhesion molecule vinculin was significantly blunted in all spaceflight samples. It influences the cell–matrix adhesion and intercellular junctions. In addition, vinculin is involved in mechanotransduction processes together with integrins at focal adhesion sites [44]. Vinculin is integrated in a network together with talin, integrins and actin. The gene expression of *VCL* was reduced in the MCS of cancer cells compared with 1*g* samples. Furthermore, the gene expression of *PXN* was reduced in spaceflight samples. These results are in agreement with the data of a recent paper [35]. The cytoskeleton, integrins and focal adhesion factors are involved in anti-apoptotic strategies, depending on the cell type [35]. MCF-7 cells exposed for 24 h to the RPM revealed a reduction in vinculin and other focal adhesion molecules as well as integrin-β1 in MCS [35]. These results support our spaceflight data for TCC. Taken together, these results show that the focal adhesion genes were decreased in spaceflight samples and focal adhesion is reduced in r-µ*g*.

#### 3.1.3. Growth Factors and Signalling Molecules Altered in Microgravity

EGF is a key factor involved in cell growth and differentiation of normal and cancer tissues and, among other factors, in tissue repair processes. The *EGF* gene expression was not significantly changed in the spaceflight samples, but in 10-day MCS, it showed a tendency to a higher expression. This finding is different compared with the results of the earlier Shenzhou-8/SimBox space mission [22]. An explanation may be the different launch and flight conditions and the stay on the ISS. An interesting human in vivo study investigated the levels of soluble factors in healthy people during a parabolic flight. Blood was taken from 12 healthy volunteers immediately before the first parabola and immediately after the last parabola. EGF decreased during the parabolic flight compared with the control period. The authors concluded that gravitational stress affects the serum concentration of factors, which are involved in numeral biological processes [45].

Interestingly, the *EGFR* gene was unaltered after 5 days but significantly downregulated after 10 days in all spaceflight samples (Figure 4D). EGFR alterations in microgravity have also been reported by other authors investigating different cancer cell types [46,47].

The *VEGFD* gene was not altered after 5 days in space. After 10 days, the *VEGFD* mRNA was slightly elevated in MCS compared with 1*g*. These data are similar to earlier results obtained after the Shenzhou-8/SimBox mission [23]. The expression of *VEGFD* was elevated in RPM AD cells and MCS cultures, as well as in the spaceflight samples, where it was even more pronounced in MCS [23]. The amount of secreted VEGF-D was stable in all groups. In spaceflight, VEGFD secretion remained nearly unaltered (Table 1). This is an interesting result. Nersita and colleagues [48] had shown decreased VEGF-D levels in patients with metastatic differentiated TC. This finding supports the hypothesis that spaceflight induces a redifferentiation of TC cells.

IL-6 and IL-8, encoded by the *IL6* and *CXCL8* genes respectively, are key cytokines involved in tumour growth, angiogenesis and metastasis [49,50,51,52]. After 5 days in space, the expression of *IL6* was significantly upregulated in AD cells but low in MCS. By contrast, after 10 days, the *IL6* expression in all spaceflight groups was low. The release of IL-6 protein was similar in all spaceflight samples and controls after 5 and 10 days. The expression of *CXCL8* was blunted in all spaceflight samples. We had demonstrated earlier that IL-6 and IL-8 are involved in gravity-sensitive signalling processes required for MCS formation [16]. It seems that this signal is attenuated over time during exposure to microgravity and that the tumour cells are driven towards a less-aggressive growth behaviour. This is in agreement with previous findings [23]. In parallel, the secretion of both cytokines remained unaltered by r-µ*g* (Table 1).

The mRNA expression of *RELA* was also reduced in all spaceflight samples and *MKI67* was significantly reduced in MCS after 5 days in space. Nuclear factor-κB (NF-κB) p65 encoded by *RELA* is involved in proliferation, progression, angiogenesis and metastasis in cancer. NF-κB RelA activation is involved in tumour growth and aggressiveness of papillary TC after tumour transformation [53]. There is also evidence that activation of the NF-κB p65 pathway plays a role in the pathogenesis of follicular TC [54]. We could show that the effect of r-µ*g* on FTC-133 cells is mainly anti-proliferative.

The Ras/Raf/MEK/ERK signalling pathway transfers extracellular signals from cell surface receptors to gene expression and regulation of various biological processes, including differentiation, proliferation, adhesion, migration and survival, among others [55]. The *ERK1* and *ERK2* mRNA levels were suppressed in spaceflight samples. This is in agreement with earlier results on other cell types. ERK1/2 signalling is important in cell apoptosis. Melanoma cells exposed to a clinostat exhibited fewer focal adhesions and altered cytoskeleton and nuclear positioning, leading to enhanced cell apoptosis via suppressing the FAK/RhoA-regulated mTORC1/NF-κB and ERK1/2 pathways [56]. Suppression of ERK signalling has also been found for macrophages cultured in space. Spaceflight and s-µ*g* conditions significantly reduce macrophage differentiation, and lead among others to changes in gene expression profiles [57]. Taken together, poorly differentiated follicular TCC in space revealed a suppression of genes involved in growth, differentiation, proliferation, focal adhesion, progression and metastasis.

### 3.2. Interaction of Genes Involved in Angiogenesis and Spheroid Formation

The interaction network of the selected genes investigated in this study revealed various interactions among them (Figure 7).

RelA is stimulated by *EGF*, *CAV1* and *ERK1/2* and inhibited by *CTGF.* An earlier study with a focus on the signalling elements IL-6, IL-8, TLN1, CTGF and NF-κB p65 had demonstrated their contribution to TC spheroid formation [58]. In r-µ*g* on the ISS, *RELA* expression was suppressed in FTC-133 cells, a phenomenon that might impact the differentiation of the TCC in space.

Spaceflight and s-µ*g* significantly reduced macrophage differentiation, mediated by the RAS/ERK/NF-κB pathway [57]. The suppression of these factors may lead to reduced activation of transcription factors involved in cell differentiation and favour 3D aggregation and other biological processes.

SRC positively influences *MKI67*, *CXCL8* and *CTGF*, which were downregulated or unaltered (*CTGF*) in FTC-133 cells cultured in space. c-Src was detected in MCF-7 MCS engineered with the RPM [59]. Spheroid formation was prevented by c-Src inhibition, an outcome that indicates the fundamental role of Src in this process. The *SRC* gene was downregulated in MCS, which may be explained by a counter-regulatory effect due to the spheroid formation in space.

EGF inhibits *CAV1* and influences a large number of genes, such as *ITGB1*, *VCL*, *COL1A1*, *IL6*, *CXCL8*, *CTGF*, *MKI67*, *ERK1*, *ERK2* and *RELA*. The low *EGF* gene expression of the FTC-133 cells cultured in space might explain the downregulation of these genes in AD and MCS. Furthermore, we detected a suppression of focal adhesion-related genes such as *VCL*, *PXN* and *TLN1*. *VCL* is influenced by EGF. The downregulation of VCL could explain the reduction in adhesion and the formation of MCS. It is known that the loss of cell–cell adhesion is involved in cancer invasion and metastasis. Vinculin influences metastasis and prognosis in several tumours [60].

Pro-inflammatory cytokines induce proliferation and differentiation of tumour cells [61]. IL-6 and IL-8 are involved in 3D formation in several tumour types [16]. IL-6 overexpression has been detected in a large number of cancer types [62]. High IL-6 levels induce tumorigenesis and are regulators of angiogenesis, invasiveness, metastasis, apoptosis and cancer cell survival [62]. IL-6 enhances cancer cell growth and VEGF synthesis in gastric cancer and malignant mesotheliomas [63,64]. Furthermore, EGFR signalling promotes induction of the IL-6 receptor controlled by mTOR and aberrant EGFR activation triggers IL-6 synthesis [65]. In addition, *EGF* is known to activate IL-6, but the *EGF* gene expression of FTC-133 cells was low after 5 days and not significantly changed after 10 days in space. This phenomenon might explain the downregulated *IL6* mRNA in AD cells and MCS after 10 days (Figure 5A), but not the increase in *IL6* mRNA in AD cells after 5 days in space. In addition, *EGFR* was downregulated after 10 days, which also contributed to a decrease in the *IL6* mRNA level of FTC-133 cells after 10 days in space.

Of course, there are some limitations of the study. Due to the very rare access to ISS experiments and the limited space available, it was only possible to examine one cancer cell line in space. We decided to carry out the CellBox-2 experiment for better comparability with FTCs, which we had previously extensively investigated in s-µ*g* in our laboratories. It must be noted here that other tumour types or other TCC (such as PTCs or ATCs) could behave differently in space. However, we plan to find out possible correlations in future space missions. Furthermore, we carried out the first ISS experiments with a single cell type, which does not represent the complex in vivo biology of tumours comprising multiple interactions of tumour cells with other (stromal) cells and with the tumour microenvironment. However, this simple setup gave us the opportunity to study the direct effects of µ*g* on tumour cells. 

**Figure 8:**
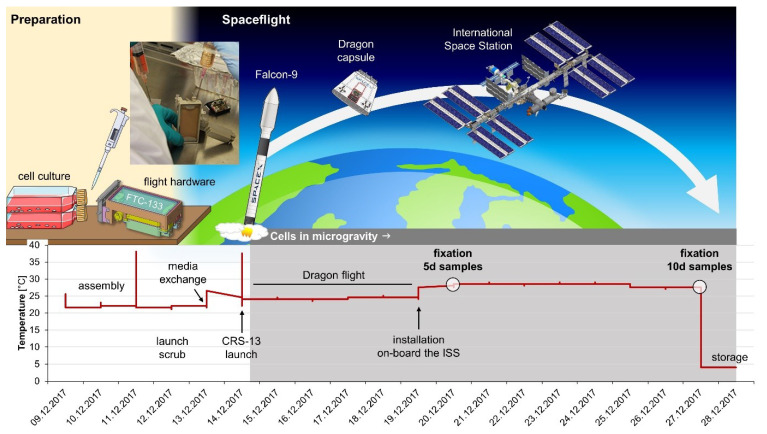
Time course and temperature profile of the Cellbox-2 spaceflight experiment on the International Space Station. An equivalent time course and temperature profile were also applied for the 1*g* ground control experiment. Parts of the figure were drawn by using pictures from Servier Medical Art, and they are licensed under a Creative Commons Attribution 3.0 Unported License (https://creativecommons.org/licenses/by/3.0/ (accessed on 20 July 2021)).

Last but not least, the cells were exposed to other stressors due to the course of the CellBox-2 mission: between handover of the samples and their installation on the ISS, cells experienced phases of suboptimal temperature (Figure 8) and different mechanical loads (rocket launch, docking manoeuvre, installation procedure), which could also impact gene and protein expression. For better comparability, we have used at least an identical temperature profile for the 1*g* controls. In addition, the impact of cosmic radiation will be addressed in future space missions. 

In summary, these data show that TCC cultured in space showed a similar secretory behaviour as TCC cultured on Earth. By contrast, the expression of genes involved in TCC proliferation, adhesion, growth and metastasis were suppressed in space samples. There are multiple indications that the cells differentiate into a less aggressive phenotype. Furthermore, we revealed a suppression of NF-κB and ERK signalling and an elevated angiopoietin 2 secretion after a 10-day stay in space. NF-κB and ERK signalling are involved in cell proliferation, differentiation, migration, senescence, apoptosis and inflammation. Angiopoietin-2 is an important proangiogenic factor implicated in mediating inflammatory processes. For these reasons, the results are of clinical relevance, but it has to be taken into account that the FTC-133 cell line is a stable platform for research on thyroid cancer but does not fully represent the situation occurring in vivo. In addition, further experiments are necessary to increase the number of spaceflight samples.

These results from space missions can be used to support conventional cancer research. New insights from spaceflight experiments support to pinpoint the mechanisms involved in cancer progression and metastasis. This new knowledge can lead to the design of novel drugs that will improve the quality of life of the patients or to the development of new preventive countermeasures. 

## 4. Materials and Methods

### 4.1. Cell Cultures

The human follicular TCC line FTC-133 is well-established and is derived from a lymph node metastasis of a follicular thyroid carcinoma of a 42-year-old man [11]. The FTC-133 cells are histologically defined as a poorly differentiated type of follicular TC and are commercially available from Health Protection Agency Culture Collections (HPACC, Salisbury, UK). The cells were cultured in RPMI 1640 medium (Life Technologies, Carlsbad, CA, USA), supplemented with 10% foetal calf serum (Sigma Aldrich, Steinheim, Germany) and 1% penicillin/streptomycin (Life Technologies, New York, NY, USA) at 37 °C and 5% CO_2_ until use for the experiment.

After the experiments, the FTC-133 cells and the cell supernatants were harvested as has been described in detail [23,58]. Collected control adherent cells from 1*g* and space samples (AD cells and MCS) were shock-frozen in liquid nitrogen and stored at −150 °C together with the supernatants for multianalyte profiling (MAP) and Luminex analyses.

### 4.2. CellBox-2 Spaceflight Experiment

The Cellbox-2 space experiment was carried out as previously described by Melnik et al. [20]. During the spaceflight, the cells were harboured in an automated hardware unit with a cell cultivation chamber consisting of polyether ether ketone (PEEK). The launch of the Falcon 9 rocket of SpaceX CRS-13 took place on 15 December 2017, 15:36:09 UTC, from the Cape Canaveral Air Force Station Space Launch Complex 40, Kennedy Space Center, Cape Canaveral, FL, USA (Figure 8). On 12 January 2018, the cells returned to Earth. The Dragon capsule spent just under a month at the ISS. The spacecraft splashed down in the Pacific Ocean at 15:37 UTC carrying equipment and science experiments.

Five days before the launch of the SpaceX rocket, 1 × 10^6^ FTC-133 cells were seeded into six hardware units. Due to a launch delay, a medium exchange had to be performed 2 days before the launch. After the launch, the dragon capsule with the cells needed 2 days to arrive at the ISS. Then, the cells were cultured at 28 °C on the ISS. Five days after the launch, the medium was changed automatically in all six hardware units. Additionally, the cells in three of the six hardware units were fixed. After another five days, the cells in the remaining three hardware units were also fixed. The fixed cells were stored at 4 °C until they returned to the laboratory.

As soon as the culture units were available, the supernatants containing detached 3D cell aggregates (MCS) were removed and centrifuged. Afterwards, the cell pellets were used for qPCR analysis, while the cell-free supernatants were forwarded to MAP analysis. Cells remaining at the bottom of the cell culture chambers (adherent cells) were scrapped off, washed and used for qPCR.

### 4.3. CellBox-2 Ground Control Experiment

Three different control experiments were performed. First, FTC-133 cells were cultured in six flight hardware units (SSCC) according to the same timeline and protocol as during the CellBox-2 space experiment, but they remained on Earth in our laboratories in Magdeburg (Figure 8). After the time course indicated in Figure 8, the cells and supernatants were harvested according to the same procedure as described in Section 4.2 for the spaceflight units.

The control experiment was performed as published earlier [21,24], but the same temperature and time profiles were applied as during the CellBox-2 space experiment (Figure 8). After 5 or 10 days, the supernatants and cells were harvested. Each supernatant of the hardware container was collected in tubes. The tubes were centrifuged at 2500× *g* for 10 min at 4 °C. Afterwards, the cells of the pellets (MCS) were used for qPCR analysis, while the cell-free supernatant was forwarded to MAP analysis. Cells adhering to the bottom of the cell culture flasks (AD cells) were collected with a cell scraper (Sarstedt, Nümbrecht, Germany) and were transferred to tubes.

### 4.4. RNA Extraction and Quantitative Polymerase Chain Reaction (qPCR)

Cells harvested from the SSCC hardware units stored either on the ISS or in a ground-based laboratory as well as cells harvested from T-25 culture flasks either mounted on an RPM or incubated in the incubator beside the RPM were washed and prepared for RNA extraction. The extraction and the subsequent qPCR were performed as recently described by Kopp et al. [18]. The same execution was applied for the ground control and spaceflight samples. The primers used for qPCR are listed in Table 2.

### 4.5. Protein Measurements by Multi-Analyte Profiling Technology

The proteins released into the supernatants of the different cultures were analysed by the company Myriad RBM (Austin, TX, USA). The analysis was performed using the Human AngiogenesisMAP^®^ v1.0 as described previously [66]. Supernatants were taken from the automated flight hardware containers at the time points indicated and stored at −80 °C until shipment to Myriad RBM.

### 4.6. Enzyme-Linked Immunosorbent Assay (ELISA) Measurements from Cell Culture Supernatants

The levels of the proteins listed in Table 1 were determined in the supernatants using commercial ELISA kits according to the manufacturer’s instructions (LifeSpan BioSciences, Seattle, WA, USA; Cusabio Biotech, Houston, TX, USA). Supernatants taken from the flight hardware were used undiluted. Ninety-six-well plates were read using a SpectraMax M2 Microplate Reader (Molecular Devices, San José, CA, USA). The data were analysed via elisaanalysis.com (Elisakit.com, Melbourne, Australia) with a 4-parameter logistic regression algorithm to generate the standard curve equation.

### 4.7. Proteomic Profiling of Plasma Analytes

Supernatants were obtained from 5-day (n = 3) and 10-day (n = 2) 1*g* samples and 5-day (n = 3) and 10-day (n = 2) microgravity samples and stored at −80 °C until shipment to Myriad RBM. Analytes derived from the Human AngiogenesisMAP^®^ v1.0 were analysed by Myriad RBM. The analyte concentrations sent by Myriad RBM were filtered and subjected to analysis of variance (ANOVA).

#### One-Way Analysis of Variance

In multianalyte profiling (MAP), 11 analytes had a sufficient concentration to be submitted to one-way ANOVA. In addition, the six analytes resulting from the enzyme-linked immunosorbent assay (ELISA) were submitted to ANOVA. Of these 17 analytes, only ANG-2 showed a significant difference in concentration at the 10% false discovery rate (FDR) level [67] (*p* = 5.39 × 10^−3^, FDR *q* = 0.097). Tukey’s honestly significant difference (HSD) post hoc test was able to narrow the significant differences to a concentration depletion of the ten-day control compared with the other three conditions (Figure 6; Table 1).

### 4.8. Pathway Analysis

To investigate the mutual regulation of genes and to visualise the localisation and interactions between proteins, we entered the relevant UniProtKB entry numbers in the Pathway Studio v.11 software (Elsevier Research Solutions, Amsterdam, The Netherlands). Graphs were generated for gene expression and protein regulation and binding. The method was described previously [18,24].

### 4.9. Statistical Evaluation 

GraphPad prism 7.01 (GraphPad Software, Inc., San Diego, CA, USA) was used to analyse the data. The Mann–Whitney U-Test was used to compare 1*g* and r-µ*g* conditions, as well as AD and MCS cells. All secretome data are presented in a table as mean ± standard deviation (SD). Differences between the groups were considered significant at *p* < 0.05 (* *p* < 0.05; ** *p* < 0.01; *** *p* < 0.001). 

## 5. Conclusions

A spaceflight to the ISS and a 10-day stay in orbit induced a relevant systemic stress for living cells. Generally, exposure to real microgravity reveals anti-proliferative effects, influences growth and alters the gene expression of a large number of genes involved in growth, adhesion, angiogenesis and metastasis. Noticeably, in response to microgravity, the FTC-133 cells revealed a suppression of NF-κB and ERK signalling. Notably, the analyses revealed that gravity impacts the expression of cytokines, the expression of genes of the focal adhesion complex and the extracellular matrix.

## Figures and Tables

**Table 1:** Overview of soluble factors secreted by FTC-133 cells during spaceflight and in ground controls. Analyte levels were determined using Myriad RBM Human AngiogenesisMAP^®^ v. 1.0 (Type = multianalyte profiling (MAP)) or enzyme-linked immunosorbent assay (ELISA) kits from LifeSpan BioSciences (Type = ELISA). For each analyte and condition, the mean is presented, with the Myriad RBM samples with analytes below the lower limit of quantitation (LLOQ) assumed as LLOQ/2. The number of samples above the LLOQ and the standard deviations are given in round brackets. Analytes with no measured value above the LLOQ for a specific condition are marked with “---”. Analysis of variance *p*-values and false discovery rate (FDR) *q*-values are listed for complete datasets.

Analytes	LLOQ (pg/mL)	5-Day µ*g* (pg/mL)	10-Day µ*g* (pg/mL)	5-Day 1*g* (pg/mL)	10-Day 1*g* (pg/mL)	*p*	FDR *q*	Type
Angiopoietin-2 (Ang-2)	33	1333.3 (3, 115.5)	1150 (2, 212.1)	1266.7 (3, 230.9)	430 (2, 70.7)	0.0054	0.097	MAP
Carbonic anhydrase 9 (CA-9)	4.2	64.3 (3, 32.5)	40 (2, 21.2)	18.3 (3, 1.53)	17 (2, 4.24)	0.11	0.64	MAP
Collagen I alpha I (BR18)	---	1934.5 (3, 1281.4)	1408.4 (2, 1748.8)	1264.1 (3, 358.3)	53.86 (2, 2.74)	0.36	0.66	ELISA
Epidermal Growth Factor (EGF)	0.74	0.5 (1, 0.23)	---	---	1 (2, 0)	---	---	MAP
Epidermal Growth Factor Receptor (EGFR)	16	32.3 (3, 6.11)	31 (2, 5.66)	21.7 (3, 8.08)	23.5 (2, 2.12)	0.23	0.66	MAP
Fatty Acid-Binding Protein, adipocyte (FABP, adipocyte)	30	87 (3, 23)	69.5 (2, 33.2)	---	---	---	---	MAP
Fibronectin (BR56)	---	606,423.3 (3, 417,312.8)	412,019.5 (2, 521,272.7)	200,447 (3, 56,096.2)	105,046.5 (2, 22,128.9)	0.37	0.66	ELISA
Granulocyte-Macrophage Colony-Stimulating Factor (GM-CSF)	5.9	9.17 (3, 2.63)	6.33 (1, 4.77)	---	5.38 (1, 3.43)	---	---	MAP
Heparin-Binding EGF-Like Growth Factor (HB-EGF)	0.7	1.43 (3, 0.31)	1.15 (2, 0.64)	1.3 (3, 0.4)	1.5 (2, 0.71)	0.88	0.88	MAP
Hepatocyte Growth Factor receptor (HGF receptor)	52	2110 (3, 1358.4)	1430 (2, 1654.6)	1240 (3, 341.8)	1150 (2, 212.1)	0.72	0.88	MAP
Insulin-like Growth Factor-Binding Protein 1 (IGFBP-1)	120	3600 (3, 556.8)	4700 (2, 1697.1)	3193.3 (3, 2174.4)	3110 (2, 3804.2)	0.86	0.88	MAP
Intercellular Adhesion Molecule 1 (ICAM-1)	570	396.7 (1, 193.4)	452.5 (1, 236.9)	443.3 (1, 274.2)	845 (2, 120.2)	---	---	MAP
Interleukin-6 (IL-6)	0.81	128 (3, 146.6)	61.5 (2, 2.12)	65.3 (3, 25)	71.5 (2, 23.3)	0.78	0.88	MAP
Interleukin-8 (IL-8)	0.63	6890 (3, 7913.4)	2505 (2, 1322.3)	2380 (3, 1796.4)	5180 (2, 2616.3)	0.66	0.88	MAP
Laminin	---	527.67 (3, 240.19)	525 (2, 237.59)	306.33 (3, 225.019)	13,393 (2, 10,875.3)	0.055	0.497	ELISA
Lipocalin-2/NGAL (BR53)	---	251.85 (3, 17.2)	243.3 (2, 12.36)	243.32 (3, 8.74)	243.3 (2, 12.36)	0.83	0.88	ELISA
Macrophage Inflammatory Protein-1 beta (MIP-1 beta)	8.2	7.07 (1, 5.14)	---	6.07 (1, 3.4)	13.5 (2, 4.9)	---	---	MAP
Macrophage Migration Inhibitory Factor (MIF)	2.1	1560 (3, 561.1)	1300 (2, 141.4)	850 (3, 130.8)	1000 (2, 282.8)	0.18	0.66	MAP
Matrix Metalloproteinase-3 (MMP-3)	10	154 (3, 104.9)	90.5 (2, 98.3)	115 (3, 27.8)	94 (2, 7.07)	0.76	0.88	MAP
Monocyte Chemotactic Protein 1 (MCP-1)	27	30.5 (2, 14.8)	22.3 (1, 12.4)	33.3 (3, 2.52)	39 (2, 8.49)	---	---	MAP
Osteopontin (BR54)	---	8524.3 (3, 197.3)	8412.0 (2, 264.5)	8399.7 (3, 188.2)	8896 (2, 420.0)	0.26	0.66	ELISA
Placenta Growth Factor (PLGF)	12	13 (1, 12.1)	---	---	---	---	---	MAP
Tenascin-C (TN-C)	1700	233,000 (3, 157,515.1)	155,800 (2, 211,000.7)	62,666.7 (3, 17,897.9)	52,000 (2, 1414.2)	0.37	0.66	MAP
Urokinase-type plasminogen activator receptor (uPAR)	250	883.3 (3, 281.8)	662.5 (1, 760.1)	793.3 (3, 83.3)	885 (2, 7.07)	---	---	MAP
VEGF-D (BR73)	---	137.74 (3, 3.52)	132.45 (2, 2.51)	135.4 (3, 2.038)	135.99 (2, 2.5)	0.31	0.66	ELISA

**Table 2:** Primers used for quantitative real-time polymerase chain reaction.

Gene	Primer Name	Sequence
*18S rRNA*	18S-F	GGAGCCTGCGGCTTAATTT
	18S-R	CAACTAAGAACGGCCATGCA
*CAV1*	CAV1-F	CCTCCTCACAGTTTCATCCA
	CAV1-R	TGTAGATGTTGCCCTGTTCC
*COL1A1*	Col1A-F	ACGAAGACATCCCACCAATCAC
	Col1A-R	CGTTGTCGCAGACGCAGAT
*CTGF*	CTGF-F	ACAAGGGCCTCTTCTGTGACTT
	CTGF-R	GGTACACCGTACCACCGAAGAT
*EGF*	EGF-F	TGCCAGCTGCACAAATACAGA
	EGF-R	TCTTACGGAATAGTGGTGGTCATC
*EGFR*	EGFR-F	TTGCCGCAAAGTGTGTAACG
	EGFR-R	GAGATCGCCACTGATGGAGG
*ERK1*	ERK1-F	ACCTGCGACCTTAAGATTTGTGA
	ERK1-R	AGCCACATACTCCGTCAGGAA
*ERK2*	ERK2-F	TTCCAACCTGCTGCTCAACA
	ERK2-R	TCTGTCAGGAACCCTGTGTGAT
*ICAM1*	ICAM1-F	CGGCTGACGTGTGCAGTAAT
	ICAM1-R	CTTCTGAGACCTCTGGCTTCGT
*IL6*	IL6-F	CGGGAACGAAAGAGAAGCTCTA
	IL6-R	GAGCAGCCCCAGGGAGAA
*CXCL8*	IL8-F	TGGCAGCCTTCCTGATTTCT
	IL8-R	GGGTGGAAAGGTTTGGAGTATG
*ITGA4*	ITGA4-F	CCAGCTGGGTAGCCCTAATG
	ITGA4-R	CCTGGCTGTCTGGAAAGTGT
*ITGB1*	ITGB1-F	GAAAACAGCGCATATCTGGAAATT
	ITGB1-R	CAGCCAATCAGTGATCCACAA
*LAMA1*	LAMA1-F	TGGGAATGGCACAGTTGTCA
	LAMA1-R	AGCCACTCTCCTCTGGGTGTT
*MKI67*	MKI67-F	TGGGGAAAGTAGGTGTGAAAGAAG
	MKI67-R	CTCCTTAAACGTTCTGATGCTCTTG
*RELA*	NFKBp65-F	CGCTTCTTCACACACTGGATTC
	NFKBp65-R	ACTGCCGGGATGGCTTCT
*PXN*	PXN-F	CATGGACGACCTCGACGC
	PXN-R	CAAGAACACAGGCCGTTTGG
*TLN1*	TLN1-F	GATGGCTATTACTCAGTACAGACAACTGA
	TLN1-R	CATAGTAGACTCCTCATCTCCTTCCA
*VCL*	VCL-F	GTCTCGGCTGCTCGTATCTT
	VCL-R	GTCCACCAGCCCTGTCATTT
*VEGFD*	VEGFD-F	TGCAGGAGGAAAATCCACTTG
	VEFGD-R	CTCGCAACGATCTTCGTCAA

## Data Availability

The data that support the findings of this study are available from the corresponding author upon reasonable request.
